# Spatial–temporal coupling coordination and interaction between digitalization and traditional industrial upgrading: a case study of the Yellow River Basin

**DOI:** 10.1038/s41598-023-44995-7

**Published:** 2023-10-21

**Authors:** Manman Wang, Xiaoya Zhu, Shi Yin

**Affiliations:** 1 College of Economics and Management, Zhengzhou University of Light Industry, Zhengzhou, 450001 China; 2 School of Politics and Public Administration, Soochow University, Suzhou, Jiangsu China; 3 College of Economics and Management, Hebei Agricultural University, Baoding, 071001 China

**Keywords:** Environmental social sciences, Socioeconomic scenarios, Sustainability, Applied mathematics

## Abstract

The realization of coupling coordination between digitalization and traditional industrial upgrading in the Yellow River Basin holds significant practical value for promoting high-quality industrial development in the region. In order to assess this coupling coordination, we utilized inter-provincial panel data from nine provinces in the Yellow River Basin, covering the period from 2011 to 2020. Through the application of a coupling coordination degree model, we calculated the degree of coupling coordination and relative development between digitalization and traditional industrial upgrading. Additionally, we conducted a spatial–temporal analysis to identify the characteristics and trends of digitalization and traditional industrial upgrading. Furthermore, we constructed a panel VAR model to examine the interactive relationship between these two factors. The findings are as follows: (1) overall, over the study period, the degree of coupling coordination between digitalization and traditional industrial upgrading in the Yellow River Basin transitioned from a disordered state to a run-in stage. The corresponding development type changed from a low steady state to a co-existence of low and medium steady states. Notably, the levels of digitalization, traditional industrial upgrading, and coupling coordination all exhibited a gradual increase, while the relative development degree declined. (2) The coupling coordination degree between digitalization and traditional industrial upgrading in the Yellow River Basin demonstrated significant regional variation. Provinces displaying a “high–high” agglomeration distribution and “low–low” agglomeration distribution were concentrated in the middle and lower reaches, as well as the upper reaches, of the Yellow River. Furthermore, there was a positive spatial autocorrelation between these regions. (3) Both digitalization and traditional industrial upgrading exhibit self-reinforcing mechanisms, and a long-term dynamic correlation exists between them.

## Introduction

The COVID-19 pandemic has served as a catalyst for a new wave of scientific and technological revolution, propelled by digitalization through the utilization of technologies such as 5G, artificial intelligence, and big data. This rapid technological advancement has accelerated the process of industrial transformation on a global scale. As digital technology penetrates and is applied in traditional industries, it gives rise to new outputs that have now become the primary driving force behind GDP growth^1^. Research indicates that digital technology plays a crucial role in driving the optimization of efficiency allocation within traditional industries. By empowering these industries with industrial technology, it enables them to enhance intra-industry labor productivity, boost enterprise research and development efforts, and improve product quality. These advancements ultimately contribute to the transformation and upgrading of traditional industries^2,3^. From a global perspective, countries around the world are actively promoting the development of the digital economy. The purpose is to effectively exploit the positive externalities brought by the development of the digital economy to facilitate the transformation and traditional industrial upgrading. For example, the United States is the first country in the world to lay out digital transformation, and continues to pay attention to the development and impact of the new generation of information technology. Over the years, it has focused on cutting-edge technologies such as big data and artificial intelligence, and has released documents such as the National Artificial Intelligence Initiative Act of 2020 (NAIIA), the National Artificial Intelligence R&D Strategic Plan, and the Federal Big Data Research and Development Strategic Plan. A policy system based on open innovation and aimed at accelerating the transformation of traditional industries has been established, which has effectively promoted the development process of digital transformation. Germany is an advocate and leader of the digital age. As early as 2013, the concept and system of Industry 4.0 based on Cyber-Physical Systems (CPS) was proposed. Furthermore, it preempts the implementation of harmonized standards and a European digital framework to maintain the global leadership of German manufacturing. In the face of the obvious lag in the digital transformation of traditional industries, the EU has gradually formed the European Industrial Digitization Strategy by integrating the industrial digitization strategies introduced by member states. The aim is to create a collaborative innovation system and foster innovative companies to ensure that the EU is a global leader in the digital transformation of industry. The China’s government has also raised the digitalization of traditional industries to a strategic height. The Government Work Report for 2020 emphasizes the need to continuously improve the integration of digital technology and manufacturing technology, and accelerate the realization of digital innovation in traditional manufacturing enterprises. The 20th CPC National Congress also makes a strategic plan to promote the high-end and intelligent traditional industries, which points out the direction for the development of China’s traditional industries in the present and the future. Hence, digitalization will become an important way to build an industrial system with core competitiveness on a global scale.

The Yellow River Basin is regarded as an important “energy basin” in China, which is the key to promoting the coordinated development of the eastern, central and western regions and driving the construction of the Belt and Road Initiative (BRI)^4^. However, owing to the lack of endogenous power, the upgrading and transformation of traditional industries in the Yellow River Basin is difficult and slow^5^. Therefore, under the dual pressure of anti-globalization and weak ability of technological innovation, how to promote the upgrading of traditional industries through digitalization and give full play to the benign coordination and interaction of the two is an important issue for the realization of high-quality development in the Yellow River Basin^6^. Moreover, the realization of ecological protection and high-quality development strategy in the Yellow River Basin is in conformity with continuous improvement of the coupling and coordination level of digitalization and traditional industrial upgrading. To cope with the dual dilemma of environmental pollution and economic lag in the Yellow River Basin, it is necessary to completely abandon the traditional extensive development model, improve the digital content of economic development, and transform from relying on resources to relying on digitalization^7,8^. However, what is the level of coupling coordination between digitalization and traditional industrial upgrading in the Yellow River Basin? In other words, how much driving force does the digitalization level of the Yellow River Basin have on the upgrading of traditional industries, and how much guiding force does the upgrading of traditional industries have on digitalization? In fact, the lack of digital driving force and the weak guiding force of traditional industrial upgrading, any of which will block the coordinated development of the two. Thus, the research on the coupling coordination and interaction between digitalization and traditional industrial upgrading in the Yellow River Basin is of fundamental significance for the national major strategy of ecological protection and high-quality development in the Yellow River Basin.

In summary, this paper comprehensively considers the existing practice of digitalization driving the organizational production transformation and industrial chain reconstruction of traditional industries, and the strong demand for digital technology in traditional industries will provide a wide range of application scenarios and market needs for digital development. In view of the great strategic position of the Yellow River Basin in China’s economic and social development, in the context of the trend of industrial transformation caused by the new generation of information technologies such as cloud computing, big data, blockchain and AI technology, the purpose of this paper is to clarify the difficulties and contradictions in the coupling process of digitalization and traditional industrial upgrading in the Yellow River Basin. The corresponding countermeasures and suggestions are given from the coupling mechanism, coupling coordination degree, relative development degree, interaction mechanism, implications and other aspects. Specifically, this paper utilizes theoretical analysis and empirical analysis to explore the internal logical relationship between digitalization and traditional industrial upgrading. Firstly, the coupling and coordination relationship between digitalization and traditional industrial upgrading in the Yellow River Basin is interpreted from the perspective of system theory. Secondly, the level of digitalization and traditional industrial upgrading in 9 provinces in the Yellow River Basin are measured. The third is to describe the spatial–temporal characteristics and evolution trend of the coupling coordination degree based on spatial correlation analysis. Fourthly, the dynamic relationship between digitalization and traditional industrial upgrading is empirically investigated in accordance with panel VAR model. Finally, it provides theoretical reference and practical policy basis for accelerating digitalization and industrial transformation and upgrading of provinces and regions in the Yellow River Basin in the new era.

Compared with the existing research, the innovative aspect of this paper is mainly reflected in the following three aspects. First, from the perspective of research content, the theoretical research on digitalization and traditional industrial upgrading is relatively insufficient. The research on the micro mechanism between the two is not in-depth, and no standardized framework has been built to discuss the relationship between the digitalization and the transformation and traditional industrial upgrading. Hence, it is hard to fully explain the mechanism of the relationship between digitalization and the traditional industrial upgrading. In addition, most of the existing studies focus on the qualitative relationship and one-way quantitative relationship between the digitalization and traditional industrial upgrading, and they are mainly regarded as independently developed but mutually influencing systems. Quantitative studies focusing on the interaction between the two from the perspective of coupling and coordination are relatively rare. Therefore, this paper explores the coupling and coordination mechanism and interaction relationship between the two from the perspective of coupling and interaction, which will enrich the theoretical research on the relationship between digital economy and industrial upgrading. Second, from the perspective of research objects, compared with the Yangtze River Economic Belt, Beijing–Tianjin–Hebei region, Northeast China and other regions, the study on the Yellow River basin is relatively inadequate. However, the upper, middle and lower reaches of the Yellow River basin span eastern, central and western China, which is an vital support for promoting high-quality development of China’s economy in the context of North–South economic differentiation and an important land area of the “Belt and Road”. Therefore, industrial transformation and development in the Yellow River Basin is the key to the major national strategy of ecological protection and high-quality development in the Yellow River Basin. This paper selects the Yellow River Basin as the research object, which not only expands the existing research scope, but also provides important basis and effective reference for traditional industries to cope with the dual challenges of industrial upgrading and digital transformation, and for government departments to formulate relevant policies. Third, from the perspective of research methods, most existing studies adopt qualitative or simple quantitative methods to unilaterally and stably evaluate the digitalization and upgrading level of traditional industries, and the evaluation results are not scientific. Based on the actual characteristics of the Yellow River Basin, this paper constructs a comprehensive index system of digitalization and traditional industrial upgrading from a multi-dimensional perspective, and examines the spatial–temporal differentiation of evaluation results from a dynamic perspective.

The remaining parts of this paper are organized as follows: Section “Literature review” briefly provides a literature review; The coupling and coordination mechanism of digitalization and traditional industrial upgrading are presented in sections “The coupling and coordination mechanism of digitalization and traditional industrial upgrading”; “Research design” displays the construction of index system of digitalization and traditional industrial upgrading in Yellow River Basin, and presentes the study method, data description and data sources; section “Empirical analysis” depicts the results of empirical analysis; and section “Conclusions and implications” summarizes the conclusions and some major policy implications.

## Literature review

### Digitization

The term “digitization” was coined by economist called Don Tapscott in 1996. At present, the research on digitalization focuses on its connotation, measurement, and economic effect. Firstly, in terms of the connotation of digitization, it can be traced back to the 1980s. With the development of digital technology and microelectronics technology, digital technology has been gradually applied to electronic equipment and media software, which has the advantages of high reliability and fast speed^9^. In addition, scholars have also expounded the connotation of digitalization from different perspectives. For example, Chmil and Dzhhutashvili^10^ put forward that digitalization of the hotel and restaurant industry includes transforming customer experience, transforming operational processes and transforming business models. Li^11^ proposed that digital economy is a new economic form produced by the development of digital technology. Naimi-Sadigh et al.^12^ believed that digitalization aims to utilize various new technologies to promote the transformation of enterprise business model, organizational structure and corporate culture. Secondly, the measurement of digitalization is generally divided into two categories. The first is the accounting method, which calculates or estimates the scale and volume of the digital economy in a certain period and region under the definition of production^13,14^. The other is to measure the development of China’s digital economy from the aspects of digital industrialization, industry digitization and digital application by constructing an index system based on entropy, grey correlation and kernel density estimation^15,16^. Third, in terms of the economic effects of digitalization, existing literatures mostly focus on the enabling effects, economic effects, transformation paths and risk management of digitalization from the macro or meso level, and some literatures discuss the economic consequences caused by the application of digital technology in micro enterprises from the perspective of a specific information technology such as digital finance, network infrastructure, Internet and artificial intelligence. For example, at the macro level, Zhou et al.^17^ proposed that the digital economy can promote economic and social development momentum, efficiency and quality change through technology empowerment, and become a new engine and new momentum to drive high-quality economic development. Qi et al.^18^ pointed out that the digital economy has significantly expanded the scale of OFDI through such mechanisms as “trade cost effect” and “institutional quality effect”. At the middle level, Zhang^19^ found that the development of the digital economy plays a significant role in promoting the digitalization, networking and intelligent transformation of the manufacturing industry and driving the industrial structure to the middle and high-end. Zhang^20^ believed that the deep integration of the digital economy and various fields of the real economy has brought about the improvement of production efficiency and the change of production mode, which has also become an important driving force for industrial transformation and upgrading. At the micro level, Wang et al.^21^ conclued that the application of digital technology significantly improved production efficiency. Yang^22^ considered that digitalization drives product innovation, process innovation, organizational innovation and business model innovation in manufacturing enterprises.

### Digitization and traditional industrial upgrading

With the development of China's digital economy, some scholars began to pay attention to the relationship between digitalization and the traditional industrial upgrading. At present, there are abundant research results on the traditional industrial upgrading, but they mainly focus on theoretical research. Specifically, from the perspective of research content, the research theme mainly focuses on the status quo, path and countermeasures of transformation. From the perspective of research, the existing researches mainly focus on the driving factors of regional traditional industrial upgrading, such as technological innovation^23–25^ and institutional environment^26,27^. In addition, some scholars have discussed the upgrading path of traditional industries such as traditional industries, resource-based industries and manufacturing industries from different industrial perspectives. From the perspective of research methods, the measurement and evaluation methods of traditional industrial upgrading are the key issues discussed by scholars. At present, there are mainly two measurement methods, one is the single indicator method. For example, the industrial structure level coefficient is usually utilized to measure the level of industrial upgrading^28,29^. The second is the comprehensive index system method. The advantage of this method is that it can build a comprehensive index system from many aspects, so as to measure and evaluate the upgrading effect of traditional industries more comprehensively^30,31^.

On this basis, scholars have studied the impact of digitalization on traditional industrial upgrading from different perspectives and reached a consistent conclusion that digitalization has a positive impact on traditional industrial upgrading. The driving effect of digitalization on the traditional industrial upgrading is mainly concentrated in two parts involving theoretical and practical research. In terms of theoretical research, scholars have explored the internal driving mechanism of digitalization to traditional industrial upgrading from the micro level. For example, Von Krogh^32^ believed that information technology enhances enterprise transformation by accelerating efficient knowledge sharing management and improving production efficiency. Miyazaki et al.^33^ and Cardona et al.^34^ concluded that the Internet stimulates the diffusion, application and innovation of industrial technology. Ivus and Boland proposed that the integration and innovation development of digital economy and traditional industries is of great significance in facilitating the transformation and upgrading of industrial structure digitalization, rationalization and greening^35^. Zuo^36^ put forward that the development of China’s digital economy promotes the upgrading of industrial structure by providing technical support, promoting the integrated development of multiple industries and promoting industrial innovation. From the perspective of the industrial chain, Kutin et al.^37^ raised that digitalization, intelligence and automation can significantly improve the overall efficiency of the industrial chain and promote the transformation and upgrading of industrial structure. Laudien and Pesch^38^ presented that traditional enterprises accelerate the transformation of traditional production factors with the help of digital technology, thus promoting the transformation of production mode, improving the allocation efficiency of production factors, and promoting the upgrading of industrial structure. Heo and Lee^39^ pointed out that there is linkage effect and diffusion effect between ICT and industry, which is helpful to push forward transformation and upgrading of dynamic manufacturing into high-tech industries. Autio^40^ found that digital technologies can effectively allocate resources at a low cost and significantly improve supply and demand allocation efficiency. In terms of practical research, scholars tend to construct panel data models to empirically investigate the impact of digitalization on the traditional industrial upgrading. For example, Shen et al.^41^ empirically concluded that the optimization and upgrading of the industrial structure of traditional manufacturing industry in Zhejiang Province is positively correlated with the construction of digital infrastructure, the level of digital technology innovation and scientific research, and the development of digital industry. Taking the new economic theory as the perspective and using static and dynamic spatial panel models, Chen et al.^42^ concluded that the digital economy can promote the upgrading of industrial structure inside and outside the provincial region. Zhang et al.^43^ detected that the coverage breadth and support service degree of digital economy have a positive effect on the transformation and upgrading of China’s economic structure. Wang and Zhang^44^ summarized through empirical research that the level of digital development can promote regional development of advantageous industries by improving local resource allocation and enhancing innovation capacity. Zhang et al.^45^ utilized static and dynamic panel regression models to estimate the impact of industrial intelligence on the upgrading and rationalization of industrial structure, and presented that industrial intelligence brought about by the application of digitalization in industries would lead to more demands for high-tech talents and affect the reallocation of production factors such as capital and labor among industries. Liu and Ji^46^ constructed a threshold effect model and concluded that digital industrialization has an inverted U-shaped incremental effect on industrial structure upgrading, industrial digitalization has a positive incremental effect on industrial structure upgrading, and digital economy has a positive incremental effect on industrial structure upgrading.

In general, the development of digitalization and traditional industrial upgrading have attracted wide attention, and the research perspective has gradually expanded, which has laid a theoretical foundation for this paper. However, on the whole, there are relatively few researches on the correlation between digitalization and traditional industrial upgrading, which makes it difficult to fully elucidate the internal mechanism of the two systems. On the one hand, at present, most researches focus on the qualitative relationship and one-way quantitative relationship between digitalization and industrial upgrading, and they are mainly regarded as systems that develop independently but interact with each other. Quantitative studies focusing on the interaction between the two from the dynamic perspective based on coupling coordination are relatively rare. On the other hand, most existing researches adopt qualitative or simple quantitative methods to static evaluate the status quo of enterprise digital transformation, industrial digitalization level and industrial transformation and upgrading, and there are relatively few researches considering the coupling coordination characteristics and dynamic evolution characteristics of evaluation results from a spatial–temporal perspective.

Given that digital integration of traditional industrial upgrading is an important support for sustainable regional development and high-quality industrial development in the Yellow River Basin. We make a contribution to the studies discussed above in several ways to provide inspiration for the practice of high-quality industrial development in the Yellow River Basin. First, we construct an evaluation index system for the evaluation of digitalization and traditional industrial upgrading in the Yellow River Basin. Second, we discuss the coupling coordination degree, coordination development process and spatial–temporal evolution characteristics of coupling coordination degree in the Yellow River Basin from the perspective of system coupling interaction. To this end, we construct the panel VAR model and empirically analyze the interaction mechanism between digitalization and traditional industrial upgrading in the Yellow River Basin.

## The coupling and coordination mechanism of digitalization and traditional industrial upgrading

In the field of economy and society, closely related economic indicators or social phenomena organically combine to form a union by promoting or restricting each other, and the form or relationship that plays a role together is the coupling in economy and society. Ecological protection and high-quality development in the Yellow River Basin cannot be achieved without the coordinated, orderly and common progress of digitalization and traditional industrial upgrading. In other words, there is a complex coupling relationship between digitalization and traditional industrial upgrading, which is a long-term and dynamic historical evolution process.

On the one hand, digitalization is the core driving force for the traditional industrial upgrading. For the development of traditional industries, digitalization takes data resources as the key element, digital technology as the core driving force, and modern information network as the main carrier. It can promote industrial transformation and upgrading by adding new production factors, changing organizational forms and innovating business models. Meanwhile, emerging industries such as the Internet of Things and artificial intelligence supported by digital technology are developing rapidly. All kinds of new digital industries highly analyze, integrate and apply digital information and knowledge to the real economy, which effectively promotes the traditional industrial upgrading^47^. Specifically, the effect of digitalization to facilitate the traditional industrial upgrading is mainly reflected in four aspects, which include the promotion of traditional industrial process upgrading, product upgrading, function upgrading and industrial ecological upgrading. First of all, digitalization can improve production technology and process, improve production efficiency and product quality, and effectively enhance the process upgrading and product upgrading. Secondly, digitalization is widely exploited in production, circulation, service, consumption and other links. It can optimize the traditional factor allocation mode and organization mode, and realize the whole process transformation of traditional industries and promote the function traditional industrial upgrading through the data as the carrier of new advanced production factors. Finally, digitalization can promote the deep integration of traditional industries and green low-carbon technologies, ameliorate the production efficiency of the whole process, reduce the energy consumption of the whole chain, achieve the double improvement of production efficiency and energy efficiency, and advance the ecological upgrading of traditional industries. On the other hand, from the reverse perspective, the traditional industrial upgrading brings new market demand, and provides a good development direction and space for digital development^48^. The opposite effect of traditional industrial upgrading on digital development is also mainly reflected in four aspects, which include ameliorating the level of digital construction, digital access, digital application and digital circulation. First of all, the diversification of traditional industrial structure and the efficient development of industry provide comprehensive development of high-tech talents and sufficient financial support for digitalization. Moreover, a reasonable industrial structure provides a good material basis for regional digital development. In such a case, the level of digital construction and digital access can be improved. Second, as the characteristics of the traditional industrial structure transition from labor-intensive to knowledge and technology-intensive, the added value of products and the output value of high-tech industries grow, and the resulting increase in returns will induce the level of digital access in the region to continue to increase. Finally, with the rationalization of the industrial structure, the flow rate of digital resources between regions is accelerated and can be rationally allocated, which reduces the waste of resources. It is beneficial to improve the level of digital circulation.

Additionally, according to spatial economics and new economic geography, regional economic development not only depends on the inherent resource endowment and input of the region, but also is affected by the economic development level of neighboring regions^49^. On the one hand, the traditional industrial upgrading in the region will produce “benchmark synergies” and “demonstration effects” in a spatial scale. They enhance the industrial development level of spatially related areas by accelerating the upgrading of industrial structure and the adjustment of energy consumption structure in surrounding areas. Some studies have pointed out that digitalization will release “technological dividends” through regional cooperation, flow of production factors and industrial linkages, which will have a positive impact on spatially related areas^50,51^. On the other hand, the degree of local protectionism, the level of social and economic development in different regions and the difference in resource input will also affect the actual spillover effect of digitalization. In the actual economic operation, the industrial development and adjustment of the region is generally at the cost of the “three high” backward industries in the neighboring less-developed regions^52^. Moreover, the inherent “exclusive competition” for material, capital and human capital in the spatially related regions will also hinder the cross-regional flow of digital resource elements to a certain extent. This situation will weaken the spillover effect of digital technology and squeeze out digital resources in related areas, and eventually lead to the unrobustness of the spillover effect of digital technology, which will have an adverse impact on the industrial development of spatially related areas^53^.

To sum up, the coupling coordination level of digitalization and traditional industrial upgrading is closely related to their mutual transformation, interdependence and mutual spillover. Furthermore, the coupling coordination level of digitalization and traditional industrial upgrading in this region will be affected by the spatial correlation of neighboring regions.

## Research design

### The construction of index system of digitalization and traditional industrial upgrading in Yellow River Basin

The coupling and coordination analysis of digitalization and traditional industrial upgrading in the Yellow River Basin needs to construct a comprehensive index of digitalization development and traditional industrial upgrading. Hence, to determine the evaluation index system of digital and traditional industrial upgrading is the premise of constructing the coupling coordination degree model. However, the evaluation standards of digitalization and traditional industrial upgrading have not been unified in the academic circle, and both are comprehensive concepts with rich connotations. The adoption of a single index cannot fully reflect their development status. This paper consequently constructs two index systems of digitalization and traditional industrial upgrading from a comprehensive perspective. On the one hand, combined with the development status of the Yellow River Basin and in light of relevant studies, the digital construction level, digital access level, digital application level and digital circulation level are selected as four first-level indicators to measure the digital level of the Yellow River Basin, and nine second-level indicators are set^54,55^. From the perspective of digital talents, funds and equipment input, the digital construction level is measured by the proportion of the number of people in higher education, R&D expenditure and the capacity of mobile phone exchange^56,57^. In addition, the Internet broadband access port reflects the investment level of network access equipment and can be utilized to characterize the level of digital access. Internet penetration rate and mobile phone penetration rate can reflect the Internet popularization degree and individual usage characteristics, and can be utilized to comprehensively examine the level of digital application^58,59^. The total length of postal routes, the number of parcels and express deliveries reflect the development level of Internet business application and the scale of online shopping industry, and they are utilized to represent the level of digital circulation. On the other hand, combined with the characteristics of traditional industries, this paper selects process upgrading, product upgrading, function upgrading and industrial ecological upgrading as four first-level indicators to measure the traditional industrial upgrading in the Yellow River Basin, and sets four second-level indicators. Among them, process upgrading is mainly reflected in the improvement of the internal production efficiency of enterprises, while the main feature of traditional industries is labor-intensive. Therefore, labor productivity can be selected to represent process upgrading^60^. Product upgrading is mainly manifested as enterprises climbing to both ends of the value chain, which specifically refers to the development of new products or upgrading of existing product functions, as well as the transformation and marketing of enterprise terminals. In this paper, sales revenue of new products and total industrial output value were utilized to characterize. Functional upgrading is reflected in the improvement of the position of traditional industries in the industrial value chain, which is manifested by the transition from low value-added links to high value-added links. The amount of profit realized by the main business income per unit is utilized to measure^61^. Industrial ecological upgrading refers to the development of traditional industries in the direction of energy conservation, high efficiency and green environmental protection. Energy consumption per unit of main business income and completed investment in industrial pollution control are expressed^62^. Details of each indicator are shown in Table 1.

**Table 1:** Weights of indexes in the subsystem of digitalization and traditional industrial upgrading in the Yellow River Basin.

Order parameter	Primary index	Secondary index	Weight
Digitization (SZH)	Digital construction level (JS)	The proportion of people with higher education	0.045
		R&D expenditure	0.076
		Mobile phone exchange capacity	0.056
	Digital access level (JR)	Internet broadband access port	0.093
	Digital application level(YY)	Internet penetration rate	0.073
		Mobile phone penetration	0.078
	Digital circulation level (LT)	Total route length (one way)	0.039
		Number of parcels	0.032
		Number of express deliveries	0.083
Traditional industrial upgrading (CSJ)	Process upgrading (GY)	Labor productivity	0.029
	Product upgrade (CP)	Revenue from new product sales	0.075
		Total industrial output value	0.065
	Function upgrade (GN)	The amount of profit per unit of main business income	0.078
	Industrial ecological upgrading (STH)	Energy consumption per unit of main business income	0.091
		Investment in industrial pollution control	0.086

### Research method

#### Global entropy method

Different from subjective weighting, analytic hierarchy and other index weighting methods, entropy method calculates index weights in consequence of the information entropy of data, which is more objective. Therefore, this method is generally utilized to assign weights to indicators. However, the traditional entropy method is based on two-dimensional data. It can only evaluate the information of multiple indicators in a particular year horizontally or a specific indicator at multiple time points longitudinally, which fails to fully reflect the information of indicators in the dual dimensions of time and space. Consequently, due to overcome the above limitations, this paper introduces the global idea, establishes the global entropy method dynamic evaluation model from the perspective of temporal and spatial variation, and evaluates the digitalization and traditional industrial upgrading respectively by utilizing the three-dimensional sequential data table of index-province-year. Taking digitization as an example, this paper evaluates it in the following seven steps.

1. Establish a global evaluation matrix.

Suppose it is necessary to evaluate the digitization level of m provinces in T years, and its evaluation index system includes n indexes. x represents the index value,i represents the province, j represents the index item, and t represents the year, where $i = 1, \ldots ,m;\;j = 1, \ldots ,n;\;t = 1, \ldots ,T$. Then there are T sheets of section data in T year. Based on the global level and in accordance with the principle of time sequence and from top to bottom, T sheets of section data are connected in series to form a $m \times T \times n$ global evaluation matrix, which is shown in Formula (1).

1 $$ X = \left( {x_{ij}^{1} ,x_{ij}^{2} ,x_{ij}^{3} , \ldots ,x_{ij}^{t} } \right)^{\prime } = \left( {x_{ij}^{t} } \right)_{m \times T \times n} $$

2. 2) Owing to eliminate the difference of dimensionality and numerical orientation of each index, the initial data in matrix X is further standardized.

2 $$ \left( {x_{ij}^{t} } \right)^{\prime } = \frac{{x_{ij}^{t} - \min \left( {x_{ij}^{t} } \right)}}{{\max \left( {x_{ij}^{t} } \right) - \min \left( {x_{ij}^{t} } \right)}} \times 99 + 1 $$

where, $\left( {x_{ij}^{t} } \right)^{\prime }$ is the standardized index value ranging from 1 to 100. $\min \left( {x_{ij}^{t} } \right)$ and $\max \left( {x_{ij}^{t} } \right)$ are respectively the minimum and maximum values of the J index of all provinces in T year.

3. Calculate the proportion of j index of province i in all provinces.

3 $$ \mu_{ij} = \frac{{\sum\limits_{t = 1}^{T} {\left( {x_{ij}^{t} } \right)^{\prime } } }}{{\sum\limits_{t = 1}^{T} {\sum\limits_{i = 1}^{m} {\left( {x_{ij}^{t} } \right)^{\prime } } } }} $$

4. Calculate the information entropy value of index j.

4 $$ Y_{j} = - k\sum\limits_{i = 1}^{m} {\mu_{ij} } \ln \mu_{ij} \begin{array}{*{20}c} {} & {k = \ln \frac{1}{mT}} \\ \end{array} $$

5. Calculate the difference coefficient of j index.

5 $$ G_{j} = 1 - Y_{j} $$

6. Calculate the weight of each index.

6 $$ \omega_{j} = \frac{{G_{j} }}{{\sum\limits_{j = 1}^{n} {G_{j} } }} $$

7. Calculate the comprehensive value of digitization.

7 $$ s_{i} = \sum\limits_{t = 1}^{T} {\sum\limits_{j = 1}^{n} {\omega_{j} x_{ij}^{t} } } $$

#### Coupling coordination degree model

Coupling refers to a dynamic correlation state in which two or more systems are closely combined and interact with each other^63^. If digitization and traditional industrial upgrading are regarded as two independent subsystems, then the associated interaction between the two subsystems can be called “digital-traditional industrial upgrading” coupling. On the one hand, the development trend of the interaction and coupling evolution within the system can be described by quantitatively measuring the coupling and coordination status of the system and describing its interaction and dynamic changes. On the other hand, it can provide a theoretical basis for distinguishing the constraints of their coupling coordination.

1. Determine the efficacy function.

Let $X_{ij} \left( {i = 1,2;\;j = 1,2, \ldots ,n} \right)$ be the order parameter of j index of subsystem i. $\alpha_{ij}$ and $\beta_{ij}$ are the upper and lower limits of the critical stable order parameters of the system respectively. $\mu_{ij}$ represents the standardized efficacy coefficient which is utilized to reflect the contribution value of variable $X_{ij}$ to the efficacy of the system. It is the satisfaction degree of the index to reach the target where $\mu_{ij} \in \left[ {0,1} \right]$. Thereinto, 0 and 1 correspond to the least and most satisfied respectively. The formula of efficiency coefficient is as follows.

8 $$ \mu_{ij} = \left\{ {\begin{array}{*{20}c} {{{\left( {X_{ij} - \beta_{ij} } \right)} \mathord{\left/ {\vphantom {{\left( {X_{ij} - \beta_{ij} } \right)} {\left( {\alpha_{ij} - \beta_{ij} } \right),\mu_{ij} > 0}}} \right. \kern-0pt} {\left( {\alpha_{ij} - \beta_{ij} } \right),\mu_{ij} > 0}}} \\ {{{\left( {\alpha_{ij} - X_{ij} } \right)} \mathord{\left/ {\vphantom {{\left( {\alpha_{ij} - X_{ij} } \right)} {\left( {\alpha_{ij} - \beta_{ij} } \right),\mu_{ij} < 0}}} \right. \kern-0pt} {\left( {\alpha_{ij} - \beta_{ij} } \right),\mu_{ij} < 0}}} \\ \end{array} } \right. $$

The “total contribution” of each order parameter in the subsystem to the “digital-traditional industrial upgrading” system is the comprehensive order parameter, which is realized in accordance with the integration method and calculated by linear weighting method^64^. Let $u_{1}$ and $u_{2}$ represent the comprehensive order parameters of digitalization and traditional industrial upgrading respectively, $u_{ij}$ represents the efficacy contribution value of order parameter j to subsystem i, and $\lambda_{ij}$ represents the corresponding weight of order parameter.

9 $$ \mu_{j} = \sum\limits_{j = 1}^{n} {\lambda_{ij} } \mu_{ij} \begin{array}{*{20}c} , & {\sum\limits_{j = 1}^{n} {\lambda_{ij} } = 1,\;i = 1,2} \\ \end{array} $$

2. The coupling degree model of digitalization and traditional industrial upgrading in the Yellow River Basin.

Referring to the concept of capacity coupling in physics, the coupling degree model of multiple system interactions can be generalized on the basis of the capacity coupling coefficient model^65^.

10 $$ C_{m} = \left\{ {{{\left( {u_{1} ,u_{2} , {\mathinner{\mkern2mu\raise1pt\hbox{.}\mkern2mu \raise4pt\hbox{.}\mkern2mu\raise7pt\hbox{.}\mkern1mu}} ,u_{m} } \right)} \mathord{\left/ {\vphantom {{\left( {u_{1} ,u_{2} , {\mathinner{\mkern2mu\raise1pt\hbox{.}\mkern2mu \raise4pt\hbox{.}\mkern2mu\raise7pt\hbox{.}\mkern1mu}} ,u_{m} } \right)} {\prod \left[ {\left( {u_{i} + u_{j} } \right)} \right]}}} \right. \kern-0pt} {\prod \left[ {\left( {u_{i} + u_{j} } \right)} \right]}}} \right\}^{{{1 \mathord{\left/ {\vphantom {1 m}} \right. \kern-0pt} m}}} $$

In summary, the coupling degree model of digitalization and traditional industrial upgrading subsystems in the Yellow River Basin is as follows.

11 $$ C = \left\{ {{{\left( {u_{1} \times u_{2} } \right)} \mathord{\left/ {\vphantom {{\left( {u_{1} \times u_{2} } \right)} {\left[ {\left( {u_{1} + u_{2} } \right) \times \left( {u_{1} + u_{2} } \right)} \right]}}} \right. \kern-0pt} {\left[ {\left( {u_{1} + u_{2} } \right) \times \left( {u_{1} + u_{2} } \right)} \right]}}} \right\}^{{{1 \mathord{\left/ {\vphantom {1 2}} \right. \kern-0pt} 2}}} $$

The interval of system coupling degree value is $C \in \left( {0,1} \right)$. When $C = 1$ and $C = 0$, the coupling degree of the digital and traditional industrial upgrading systems in the Yellow River Basin is the peak value and the valley value, which means that the two subsystems are in benign resonance coupling and independent state respectively.

3. The coupling coordination degree model of digital and traditional industrial upgrading systems in the Yellow River Basin.

The coupling degree is designed to determine the degree and time interval of coupling action. It should be noted that a single index of coupling degree is utilized to reflect the degree of coordinated development of the whole system, and cannot be utilized to compare the coupling effect of sub-industry subsystems. Due to evaluate the coordination degree of interactive coupling between digitalization and traditional industrial upgrading systems in different regions of the Yellow River Basin, the coupling coordination degree model is further constructed as follows.

12 $$ \left\{ {\begin{array}{*{20}c} {D = \left( {C \times T} \right)^{{{1 \mathord{\left/ {\vphantom {1 2}} \right. \kern-0pt} 2}}} } \\ {T = au_{1} + bu_{2} } \\ \end{array} } \right. $$

In Formula (12), C and D represent coupling degree and coupling coordination degree respectively. T stands for comprehensive harmonic index, which reflects the overall synergistic effect of digital and traditional industrial upgrading systems. In addition, a and b are undetermined coefficients.

#### Relative development model

Owing to further clarify the degree of restriction and interference between digitalization and traditional industrial upgrading, the “steady state” theory of ecology is utilized for reference to measure the relative development degree of nine provinces in the Yellow River Basin with the relative development model as follows.

13 $$ \lambda = {{U_{2} } \mathord{\left/ {\vphantom {{U_{2} } U}} \right. \kern-0pt} U}_{1} $$

Thereinto, λ is the relative development degree (or interference degree). When λ ∈ (0,0.9), it means that traditional industrial upgrading lags behind that of digitalization. When λ ∈ (0.9, 1.1), it means that the digitalization level is equivalent to traditional industrial upgrading, and the synchronous development of the two is basically realized. When λ ∈ (1.1, ∞), it indicates that the digitalization level lags behind the traditional industrial upgrading level.

Based on the above mentioned, the coupling coordination degree index D is utilized to represent the coupling coordination state between digitalization and traditional industrial upgrading in the Yellow River Basin, and the relative development degree λ is utilized to represent the development type of the two systems in the coupling coordination process. The coupling coordination state of digital and traditional industrial upgrading can be divided into four stages including low level coupling, antagonistic stage, run-in stage and high level coupling stage^66^. Moreover, the development types between digitalization and traditional industrial upgrading in the Yellow River Basin are divided into four stages containing disordered state, low-steady state, medium-steady state and high-steady state with a total of ten types as shown in Table 2.

**Table 2:** Grade evaluation criteria for coupling coordination degree.

Coupling phase	Coupling coordination degree	Relative development degree	Type	Coupling coordination feature	Development phase
Low level coupling	$0 \le D \le 0.3$		I	With the disorderly development of digitalization, the upgrading of traditional industries has received less attention	Disordered state
Antagonistic stage	$0.3 < D \le 0.6$	$0 < \lambda \le 0.9$	II	The speed of digital development is faster than that of upgrading and optimizing traditional industries	Low-steady state
		$0.9 < \lambda \le 1.1$	III	The speed of digital development is moderate, and the upgrading of traditional industries can be optimized in the short term	Low-steady state
		$\lambda > 1.1$	IV	The speed of digital development is slow, and the upgrading of traditional industries can be optimized in the short term	Low-steady state
Run-in stage	$0.6 < D \le 0.8$	$0 < \lambda \le 0.9$	V	The digital development speed is fast, the system boundary run-in is rapid	Medium-steady state
		$0.9 < \lambda \le 1.1$	VI	The digital development speed is moderate, and the system boundary is dynamically running-in	Medium-steady state
		$\lambda > 1.1$	VII	The speed of digital development is slow, and the system boundary is slow to run in	Medium-steady state
High level coupling	$0.8 < D \le 1$	$0 < \lambda \le 0.9$	VIII	Digitalization advanced development, traditional industrial upgrading can be basically optimized	High-steady state
		$0.9 < \lambda \le 1.1$	IX	The coordinated development of digitalization and the upgrading of traditional industries is ideal	High-steady state
		$\lambda > 1.1$	X	The speed of digital development has slowed down, and the upgrading of traditional industries can be basically optimized	High-steady state

#### Spatial correlation analysis

The global Moran’s I index reflects the degree of spatial autocorrelation of the research object. If Moran’s I > 0 or Moran’s I < 0, it proves the existence of spatial positive correlation or spatial negative correlation. In addition, if Moran’s I = 0, it is random.

14 $$ I = \frac{n}{{\mathop S\nolimits_{0} }}\frac{{\sum\limits_{i = 1}^{n} {\sum\limits_{j = 1}^{n} {\mathop w\nolimits_{i,j} \mathop z\nolimits_{i} \mathop z\nolimits_{j} } } }}{{\sum\limits_{i = 1}^{n} {\mathop z\nolimits_{i}^{2} } }} $$

$w_{i,j}$ is the spatial weight between elements i and j, $z_{i}$ is the deviation of element i, and n is the total number of elements. $S_{0}$ is the weight of the whole set. The details are as follows.

15 $$ \mathop S\nolimits_{0} = \sum\limits_{i = 1}^{n} {\sum\limits_{j = 1}^{n} {\mathop w\nolimits_{i,j} } } $$

The value formula of Z-value test is as follows.

16 $$ Z = \frac{{Moran^{\prime}sI - E\left( I \right)}}{{\sqrt {VAR\left( I \right)} }} $$

17 $$ E\left( I \right) = \frac{ - 1}{{\left( {n - 1} \right)}} $$

18 $$ VAR(I) = E(I^{2} ) - E(I)^{2} $$

#### Construction of panel VAR model

The panel VAR model was proposed by Holtz-Eakin^67^ and optimized by Lutkepohl^68^. It regards all research variables and their lag terms as explained variables and explanatory variables respectively, which has advantages in the interaction between real response variables. In addition, it provides a theoretical basis for the study of the bidirectional dynamic correlation between digitalization and traditional industrial upgrading in the Yellow River Basin. Combined with the research content, the model is set as follows.

19 $$ x_{it} = \sum\limits_{j = 1}^{p} {m_{j} x_{it - j} } + \alpha_{i} + \beta_{t} + \mu_{it} $$

20 $$ y_{it} = \sum\limits_{j = 1}^{p} {n_{j} y_{it - j} } + \lambda_{i} + \gamma_{t} + \delta_{it} $$

where $x_{it} = \left[ {CSJ_{it} ,JS_{it} ,JR_{it} ,YY_{it} ,LT_{it} } \right]$ in Formula (19), endogenous variables are traditional industrial upgrading, digital construction level, digital access level, digital application level and digital circulation level. Meanwhile, where $y_{it} = \left[ {SZH_{it} ,GY_{it} ,CP_{it} ,GN_{it} ,STH_{it} } \right]$ in Formula (20), endogenous variables are digitalization level, process upgrade, product upgrade, function upgrade and industrial ecological upgrade. In addition, $m_{j}$ and $n_{j}$ are matrix of coefficients. $x_{it - j}$ and $y_{it - j}$ are the j-order lag terms of $x_{it}$ and $y_{it}$, respectively. Both $\alpha_{i}$ and $\lambda_{i}$ are individual effect variables reflecting regional heterogeneity. Both $\beta_{t}$ and $\gamma_{t}$ are time effect vectors representing the specific impact of each period. Both $\mu_{it}$ and $\delta_{it}$ are perturbations. i represents the provinces in the Yellow River Basin, and t represents the year.

### Data description and data sources

In this paper, the panel data of 9 provinces in the Yellow River Basin during 2011–2020 are taken as samples, and the relevant data are mainly from China Statistical Yearbook (2012–2021) and the Statistical Yearbook of 9 provinces in the Yellow River Basin. Due to ensure the accuracy and authoritativeness of the data, linear interpolation method were capitalized to process the indicators with less and more data missing in the statistical yearbook. The key statistics of the key variables are shown in Table 3.

**Table 3:** Descriptive statistics.

Variable	Mean	Std. Dev.	Min	Max
1. SZH	31.963	15.856	7.810	72.370
2. CSJ	26.595	23.242	1.790	94.130
3. JS	0.487	0.350	0.033	1.468
4. JR	3.321	3.198	0.124	12.391
5. YY	0.827	0.346	0.156	1.654
6. LT	0.369	0.296	0.031	1.335
7. GY	8.012	1.035	6.171	9.358
8. CP	4.235	5.865	0.235	23.462
9. GN	9.436	7.741	0.254	25.371
10. STH	7.332	6.334	0.257	25.652

## Empirical analysis

### Analysis of measurement results

#### The measuring results analysis of composite index

**Figure 1:**
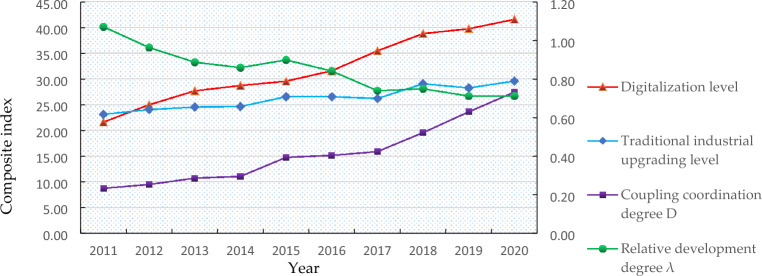
Time series trend of composite index.

Combined with the previous model construction and the classification criteria of coupling coordination stage in Table 2, the stata software is utilized to calculate the digitalization level, traditional industrial upgrading level index, the coupling coordination degree of digitalization and traditional industrial upgrading, and the relative development degree of digitalization and traditional industrial upgrading in the Yellow River Basin during 2011–2020. The measurement results are shown in Table 4. Meanwhile, it describes the time series trend chart of each composite index for a more intuitive comparative analysis in Fig. 1. Specifically, the evolution rate of digitization level in the Yellow River Basin was positive increasing from 21.60 in 2011 to 41.57 in 2020 with an increase of 93.06%. The overall upward trend was obvious. The traditional industrial upgrading in the Yellow River Basin showed a steady improvement trend. Except for a slight decline in 2016–2017 and 2018–2019, there was no significant fluctuation on the whole, which increased by 27.95% from 23.11 in 2011 to 29.57 in 2020. The exponential curve corresponding to coupling coordination degree increased steadily, while the relative development degree decreased gradually. It was in the disordered development stage of low level coupling coordination during 2011–2014. In general, it was in the low steady state of antagonism during 2015–2018, and it was in the high steady state of run-ins during 2019–2020. It can be seen that the coupling coordination degree between digitalization and traditional industrial upgrading in the Yellow River Basin is constantly optimized, while the latter lags behind the former and the two do not match.

**Table 4:** Evaluation results of digitalization and traditional industrial upgrading in the Yellow River Basin.

Year	Digitalization level	Traditional industrial upgrading level	Coupling coordination degree D	Relative development degree λ	Type	Development stage
2011	21.60	23.11	0.23	1.07	Low level coupling coordination	Disordered state
2012	24.97	24.03	0.25	0.96	Low level coupling coordination	Disordered state
2013	27.67	24.53	0.29	0.89	Low level coupling coordination	Disordered state
2014	28.70	24.63	0.29	0.86	Low level coupling coordination	Disordered state
2015	29.55	26.54	0.39	0.90	Antagonistic stage	Low steady state
2016	31.57	26.54	0.40	0.84	Antagonistic stage	Low steady state
2017	35.46	26.19	0.42	0.74	Antagonistic stage	Low steady state
2018	38.80	29.05	0.52	0.75	Antagonistic stage	Low steady state
2019	39.74	28.24	0.63	0.71	Run-in stage	Medium-steady state
2020	41.57	29.57	0.73	0.71	Run-in stage	Medium-steady state

Traditionally, the 18th National Congress of the Communist Party of China (CPC) in 2012 attached great importance to the development of the digital economy and elevated it to a national strategy. The 19th CPC National Congress in 2017 put forward a grand blueprint for building a “digital China” and a “smart society”. Under the great attention of the China’s government and a series of policy guidance, China’s digital economy has developed rapidly and ranked second in the world for a long time. The 20th CPC National Congress in 2022 further proposed the task of “Accelerating the development of the digital economy, promoting the deep integration of the digital economy and the real economy, and creating an internationally competitive digital industrial cluster”. With the rapid penetration of digital technology in various industries, industrial digitalization has gradually become the main engine of digital economy development and continues to consolidate its dominant position. Along with the continuous development of regional economy, China attaches more and more importance to the development of the Yellow River Basin. The phenomenon of “heavy industry and heavy energy” in the traditional industries of the Yellow River basin is serious, which hinders the high-quality development of the basin. Therefore, the ecological protection and high-quality development of the Yellow River Basin was subsequently elevated to a major national strategy in 2019, which clearly proposes to vigorously advance the construction of new infrastructure such as digital information, enhance the penetration rate of industrial Internet, artificial intelligence and big data to traditional industries, and foster the green transformation, intelligent upgrading and digital empowerment of the advantageous manufacturing industries in the Yellow River Basin. In line with the trend of digital development, the provinces and regions of the Yellow River Basin focus on digital technology infrastructure construction, strengthen the integrated development of digital technology, improve the digital technology governance system, scientifically layout digital technology to promote ecological protection, and promote the accelerated integration and development of industrial digitalization. However, the problem of unbalanced and inadequate development in the Yellow River basin is prominent^69^. For example, the economic ties between provinces are not close, the regional division of labor coordination and cooperation awareness is poor, and the level of economic development is uneven, which seriously restricts the nine provinces along the Yellow River to fully realize high-quality development^70^. Furthermore, the industries of the provinces in the Yellow River basin lack of emerging industrial clusters with strong competitiveness, which are dominated by energy, chemical industry, raw materials, agriculture and animal husbandry. Moreover, traditional enterprises such as coal, chemical industry and smelting have large stocks and prominent problems of low quality and efficiency. Therefore, there are many deficiencies in the digital support capacity of the Yellow River Basin, and the degree of social informatization and the development level of digital economy in the basin are still at the downstream level of the country. At the same time, there are still many problems in the construction process of “digital government” in the Yellow River Basin, such as insufficient top-level design force, low degree of intensification of information infrastructure, backward platform technology, weak basic support ability, and insufficient integration of online and offline, which leads to the failure of digitalization to effectively play the driving role of the optimization and reallocation of traditional production factor resources^30^, and the lag in the upgrading and development of traditional industries is in the digital development.

#### The coupling and coordination process of digitalization and traditional industrial upgrading in the Yellow River Basin

As shown in Table 5, the coupling coordination degree between digitalization and traditional industrial upgrading in the Yellow River Basin gradually experienced a transformation process of “disordered state → disordered state co-existing with low steady state → low steady state → low steady state co-existing with medium steady state”. At present, it was still in the stage of low steady state co-existing with medium steady state. Moreover, t was in the disordered development stage in 2011, which was in the low-level coupling stage. The D value of the coupling coordination degree of the nine provinces in the Yellow River Basin is generally low, and the λ value of the relative development degree is greater than 1.1, which indicates that the digital development level lags behind the traditional industrial upgrading level. In general, digitalization is developing disordered at this stage, and the traditional industrial upgrading has not received key attention. From 2012 to 2015, the Yellow River Basin was in the disordered state co-existing with low steady state. In this stage, Sichuan (2013), Shaanxi (2013), Shanxi (2013) and Shandong (2012) gradually got rid of disordered state and entered the stage of low steady state. These provinces have a relatively advanced level of economic development and industrialization, a deep foundation for industrial development, and perfect digital infrastructure, which provides good conditions for digital development, and the dividends brought by the digital economy can be effectively released. Therefore, the coupling and coordination degree of digitalization and traditional industrial upgrading is relatively high. On the contrary, the location conditions and digital foundation are relatively weak, the degree of modernization of the economic system is not high, and the advantages of industrial digital transformation are not obvious^71^. The digital economy has failed to give full play to the advantages of dividends, and the coupling and coordination degree of digital and traditional industrial upgrading has a large room for improvement. Except for Gansu, the coupling coordination degree of the other 8 provinces was in the low steady state of the antagonistic stage until 2016. At this stage, the coupling degree of digitalization and traditional industrial upgrading gradually increased, the relative development degree began to decrease, and the speed of digitalization process increased. Since 2016, all the nine provinces in the Yellow River Basin have been out of the disorder stage, the coupling degree of digitalization and traditional industrial upgrading has continued to increase, the relative development degree has continued to decrease, and the speed of digitalization process has gradually increased. From 2017 to 2020, it will be in the stage of low steady state co-existing with medium steady state. At this stage, the overall coupling coordination degree continues to rise and the relative development degree continues to decrease. Among them, Sichuan and Shandong are outstanding, and their coupling coordination degree breaks through 0.6 to enter the running-in stage, while the other provinces are still in the low-steady and antagonistic stage at this stage. It can be seen that the coupling coordination degree of digitalization and traditional industrial upgrading in the Yellow River Basin is gradually optimizing, and this phenomenon is obviously reflected in all provinces. In addition, the coupling coordination degree and relative development degree of each province showed a steady increase and a gradual decrease trend respectively. It is expected that all provinces will get rid of the current situation of coexistence of low steady state and medium steady state, and turn to the medium steady state or high level coupling of high steady state stage.

**Table 5:** The development stage of coupling coordination.

Province	Qinghai	Sichuan	Gansu	Ningxia	Inner Mongolia	Shaanxi	Shanxi	Henan	Shandong
2011	I	I	I	I	I	I	I	I	I
2012	I	I	I	I	I	I	I	I	IV
2013	I	II	I	I	I	II	II	I	IV
2014	I	II	I	I	I	II	II	I	IV
2015	I	II	II	IV	I	II	II	II	IV
2016	III	II	II	IV	I	II	II	II	IV
2017	III	VI	II	IV	II	IV	III	II	VII
2018	III	VI	II	IV	II	IV	III	II	VII
2019	III	VI	II	IV	II	IV	III	III	VII
2020	III	VI	II	IV	II	IV	III	III	VII

Notably, it is worth noting that this measure result has some practical significance. According to the Research Report on the Development of Digital New Economy in the Yellow River Basin released by the China Institute of Electronic Information Industry Development in May 2022, the development of digital new economy in the Yellow River Basin shows a steady growth trend. Shandong ranks in the forefront of various indexes, and the overall development level of digital economy is the most prominent. Going back to the source, Shandong is a big province of real economy with traditional industries account for up to 70% from the perspective of industrial structure. Due to promote the traditional industrial upgrading, Shandong aims at the direction of intelligence and high-end, promotes the integration of industrial Internet into the park, carries out the digital transformation action of traditional industries, supports the development zone to vigorously develop digital core industries, and nurtures a number of provincial demonstration bases for new industrialization industries, such as the Shandong Yellow River Digital Economy Industrial Park. The rapid development of digitalization has made it the second industrial Internet demonstration zone in China after Shanghai. Shandong has 29 state-level professional platforms, 31 platform innovation navigation application cases, and 4 “digital navigation” enterprises, all of which rank first in the country in April 2023. Moreover, the application rate of industrial cloud platform is 63.3% and the comprehensive digitization rate of key business links is 70.4% in Shandong, which are among the top three in the country. In contrast, the traditional industrial structure of Sichuan Province exceeds 70%. In order to set a benchmark for China’s digital economy innovation and development, strengthen the digital economy, and effectively support high-quality development, the National Development and Reform Commission authorized six provinces (Hebei (Xiongan New Area), Zhejiang, Fujian, Guangdong, Chongqing, and Sichuan) as national digital economy innovation and development pilot zones in 2019. Sichuan is one of them and the only one among the nine provinces in the Yellow River basin. In response to the call of the state, Sichuan continued to promote the high-end of advantageous industries, the new type of traditional industries and the scale of emerging industries, and the final results were remarkable. For example, the industrial Internet platform in the electronic information industry cluster area of Sichuan has driven the regional economic increase of more than 20 billion yuan. Sichuan’s digital economy will exceed 2 trillion yuan and account for 40% of GDP until 2022. The integration of industrialization and information technology is developing rapidly, and the average annual growth rate of development ranks second in China. The numerical control rate of key processes and the penetration rate of digital R & D design tools reached 54.6% and 80.9% respectively.

### Spatial–temporal evolution of coordination degree between digitalization and traditional industrial upgrading in the Yellow River Basin

#### The temporal evolution characteristics of the coordination degree between digitalization and traditional industrial upgrading in the Yellow River Basin

Furthermore, the non-parametric kernel density estimation method based on kernel function is employed to investigate the dynamic change trend of the coordination degree between digitalization and traditional industrial upgrading in the Yellow River Basin. The basic principle is as follows: Let X_1_, X_2_, …, X_n_ follows the same distribution, and its probability density f(x) must be obtained by sample estimation. Sample empirical distribution function F(X) is shown in Formula (21).

21 $$ F\left( x \right) = \frac{1}{n}\left\{ {x_{1} ,x_{{2}} , \ldots ,x_{n} } \right\} $$

The probability density estimation of fixed bandwidth is shown in Formula (22).

22 $$ f\left( x \right) = \frac{{\left[ {F\left( {x + h_{n} } \right) - F\left( {x - h_{n} } \right)} \right]}}{2h} = \int_{{x - h_{n} }}^{{x + h_{n} }} {\frac{1}{h}K\left( {\frac{t - x}{{h_{n} }}} \right)} dF_{n} \left( t \right) = \frac{1}{{nh_{n} }}\sum\limits_{i = 1}^{n} {K\left( {\frac{{x - x_{i} }}{{h_{n} }}} \right)} $$

where n is the number of samples, h is the bandwidth, and $K\left( \cdot \right)$ is the kernel function. In order to maximize the fitting effect, the commonly utilized Epanechnikov kernel function was selected, and the data-based automatic bandwidth was further selected according to the principle of minimum mean square error^72^.

**Figure 2:**
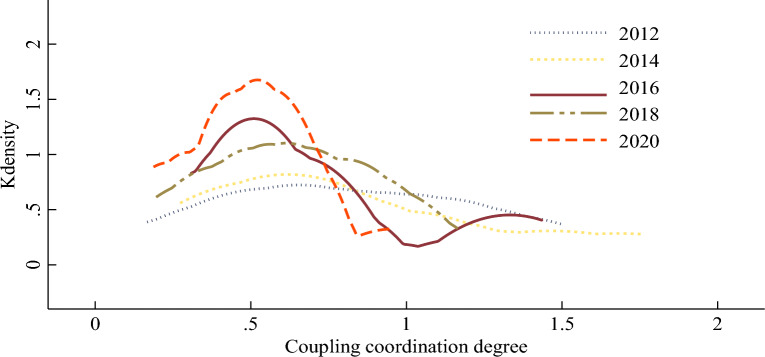
The kernel density estimation of coordination degree between digitalization and traditional industrial upgrading in the Yellow River Basin.

In this paper, five years (2012, 2014, 2016, 2018 and 2020) are selected as investigation profiles, and the 10-year data of nine provinces in the Yellow River Basin are decomposed into four stages. It depicts the kernel density curve of the coordination degree between digitalization and traditional industrial upgrading in the Yellow River Basin in Fig. 2. It can be seen that the dynamic evolution of the coordination degree distribution between digitalization and traditional industrial upgrading in the Yellow River Basin from 2011 to 2020 has a distinct feature. First of all, the coordination degree between digitalization and traditional industrial upgrading in the Yellow River Basin presents an obvious “right-skewed” distribution on the whole, which indicates that there are a few provinces with high coordination degree. Secondly, with the continuation of time, the peak height shows an obvious upward trend, the peak moves slightly to the left, the extension degree of the right tail continues to decrease, and its corresponding width narges, which indicates that the coordination degree of digitalization and traditional industrial upgrading continues to rise, and the inter-provincial gap has a trend of narrowing. Finally, the coordination degree of digitalization and traditional industrial upgrading presents a bimodal distribution in some years, which indicates that the overall development of the coordination degree of digitalization and traditional industrial upgrading is scattered, and the inter-provincial development is not coordinated and polarized. To find out its cause, the Yellow River basin runs through the east, middle and west of China. Some provinces are far apart, which results in great differences in economic strength and digitalization level within the basin. In general, the economy of the Yellow River Basin shows a pattern of “strong downstream and weak upstream”^73^. In 2020, the GDP of the nine provinces in the Yellow River Basin will reach 25.39 trillion yuan, of which the regional GDP of Henan and Shandong in the middle and lower reaches of the Yellow River basin account for more than 50 percent, while the GDP of the upper Yellow River regions such as Qinghai, Gansu, Ningxia and Inner Mongolia excluding Sichuan only account for 13.11 percent. The inter-provincial digital economy in the Yellow River Basin also has the characteristics of unbalanced development of “strong in the east and weak in the west” and “strong in the south and weak in the north”. As early as 2018, the digital economy scale of Shandong exceeded 2 trillion yuan, while the digital economy scale of Ningxia and Qinghai was only between 60 and 90 billion yuan.

#### The spatial evolution characteristics of coordination degree between digitalization and traditional industrial upgrading in the Yellow River Basin

Due to more intuitively observe the spatial characteristics of the coupling coordination degree in each region of the Yellow River basin, this paper draws the global Moran’s I index table and the local Moran’s I index scatter chart of the nine provinces of the Yellow River Basin on the basis of the economic distance weight matrix. According to Table 6, the Moran’s I index of the coordination degree between digitalization and traditional industrial upgrading in the Yellow River Basin during the sample observation period is all positive, and the empirical results show that there is a positive spatial correlation between digitalization and traditional industrial upgrading coordination in the Yellow River Basin. The coupling coordination level of digitalization and traditional industrial upgrading among provinces is closely related to the coupling coordination level of neighboring regions, which is shown as a relatively stable spatial clustering feature.

**Table 6:** The global moran’s I index.

Index	2011	2012	2013	2014	2015	2016	2017	2018	2019	2020
Moran’s I	0.064	0.057	0.065	0.028	0.021	0.033	0.058	0.046	0.065	0.055
E(I)	− 0.125	− 0.125	− 0.125	− 0.125	− 0.125	− 0.125	− 0.125	− 0.125	− 0.125	− 0.125
Z	2.216	2.159	2.261	1.824	1.301	1.848	2.193	2.016	2.243	2.128
P	0.013	0.015	0.012	0.034	0.097	0.032	0.014	0.022	0.012	0.017

**Figure 3:**
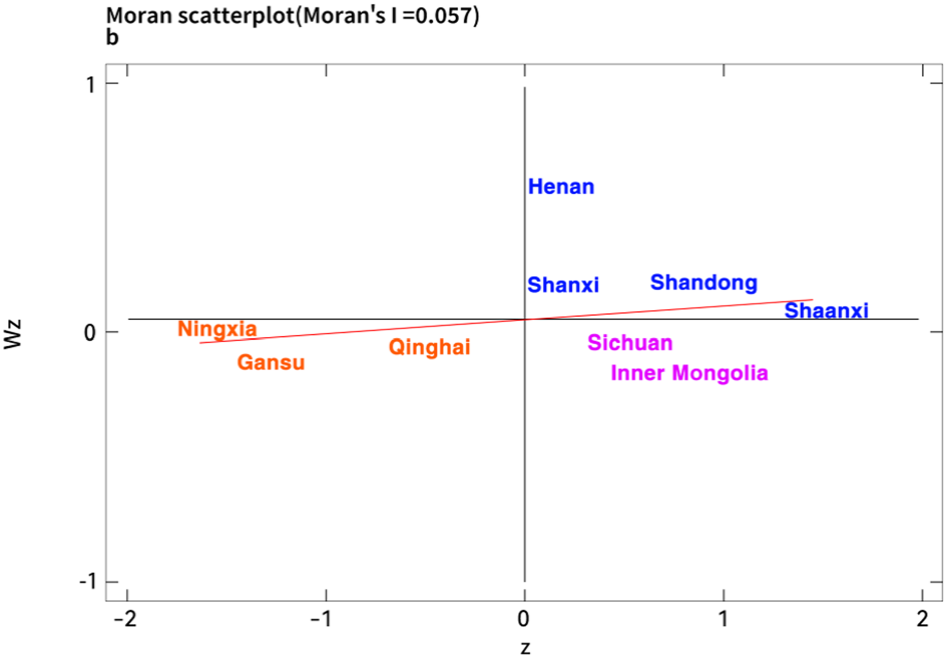
The moran index scatter plot of coupling coordination degree in the Yellow River Basin in 2012.

**Figure 4:**
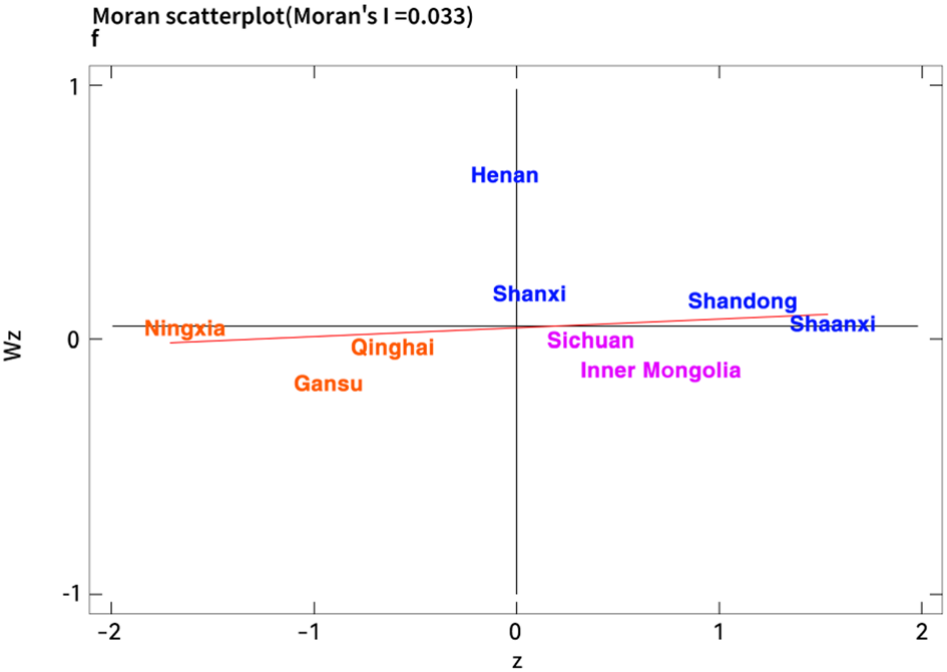
The moran index scatter plot of coupling coordination degree in the Yellow River Basin in 2016.

**Figure 5:**
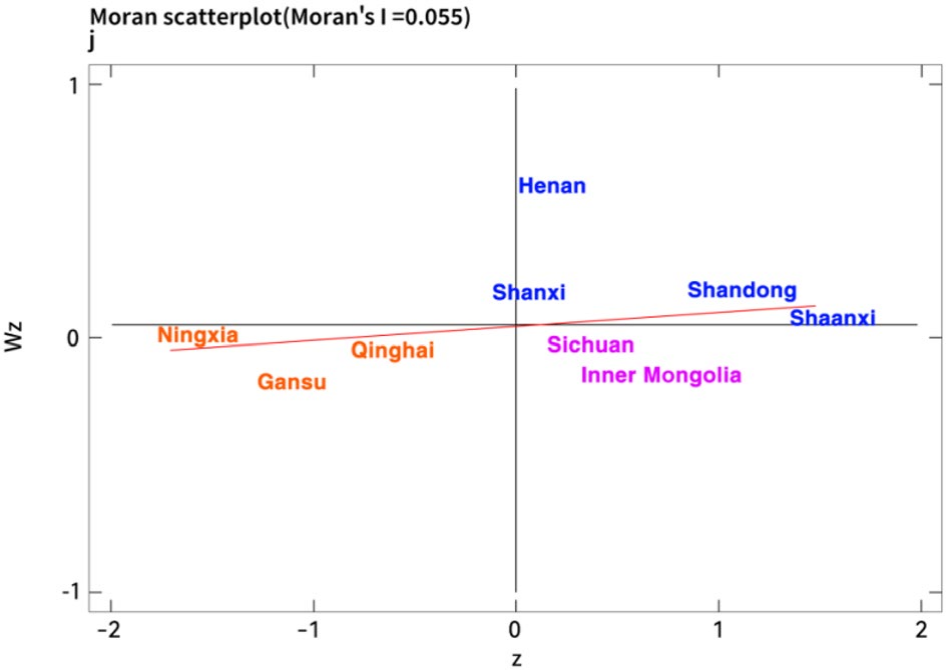
The moran index scatter plot of coupling coordination degree in the Yellow River Basin in 2020.

Furthermore, limited by space, this paper focuses on analyzing the spatial agglomeration state of coupling coordination degree in the Yellow River Basin in 2012, 2016 and 2020 corresponding to Figs. 3, 4 and 5. Concretely, the provinces of Henan, Shanxi, Shandong and Shaanxi in the first quadrant are concentrated in the middle and lower reaches of the Yellow River, which showes a “high-high” (H–H) agglomeration distribution situation. However, the provinces of Ningxia, Gansu and Qinghai in the third quadrant are mainly concentrated in the upper reaches of the Yellow River, which showes a “low-low” agglomeration (L–L) distribution. Specifically, the coupling coordination degree between digitalization and traditional industrial upgrading in the Yellow River Basin has obvious spatial difference. Due to the superior geographical conditions, economic advantages and technical conditions, there is a large spatial connection and strong spatial effect among the regions in the lower Yellow River with high level coupling, which leads to a high-value agglomeration state in the transformation and traditional industrial upgrading driven by digitalization. Nevertheless, limited by location conditions and economic system, the digital technology level of the upstream region is not mature, industrial technology lags behind and structural transformation is hindered^74^. These problems lead to the lack of internal impetus for regional industrial upgrading, and its digital development speed is lower than that of traditional industrial upgrading. Hence, the low level coupling region also has a certain spatial autocorrelation effect which is relatively small.

### Interactive mechanism between digitalization and traditional industrial upgrading in the Yellow River Basin

#### Regression results of stationarity test, co-integration test and lag order selection

A prerequisite for the application of panel VAR model is the stationarity and co-integration relationship between variables. Therefore, this paper selected Fisher-ADF test and LLC test to test the common and individual unit roots. In addition, Fisher ADF and Fisher PP tests were also used to make the test results more accurate. The test results in Table 7 show that all ten variables are first-order unitary. On this basis, Kao test is continued to verify the co-integration relationship between variables. The panel co-integration test results of the two models are listed in Table 8, and the results show that there is a co-integration relationship between variables at the significance level of 1% and 5%. The panel VAR model can be consequently selected to examine the interactive relationship between digitalization and the traditional industrial upgrading. In addition, the length of the lag order is related to the accuracy of the estimated results, and the determination of the lag order is the second prerequisite for the application of the panel VAR model. In particular, the loss of degrees of freedom will bring significant deviation to the empirical results under the small samples in this paper. It lists the test results of lag order according to the commonly exploited AIC, BIC and HQIC criteria in Table 9, and the results show that the selection of second-order lag panel VAR model is more reasonable.

**Table 7:** Test results of panel unit root test.

Variables	LLC	IPS	Fisher ADF	Fisher PP
DSZH	− 6.786**	− 2.986***	− 6.4537***	163.5577***
DCSJ	− 11.564***	− 1.569***	− 2.5696***	32.8620**
DJS	− 6.704*	− 1.673***	− 1.5342*	185.5129***
DJR	− 7.241***	− 1.815	− 1.2037***	41.9307***
DYY	− 6.588***	− 1.831	− 2.3558***	45.1995***
DLT	− 8.714***	− 2.395***	− 5.2931***	60.4260***
DGY	− 15.283***	− 3.989***	− 7.4613***	41.8803***
DCP	-11.781***	− 3.293***	− 3.0325***	72.6837***
DGN	− 15.283***	− 3.989***	− 7.4613***	41.8803***
DSTH	− 6.937***	− 1.863*	− 4.2726***	87.7718***

Note: ***, **, * denote significant at the 1%, 5% and 10% levels respectively.

**Table 8:** Test results of panel co-integration test.

Test statistics	DF	ADF
Model (19)	− 1.3047**	− 2.8337***
Model (20)	− 1.0359***	− 1.4943**

Note: The trend forms of the test model are intercept term and indeterminate trend.

Note: ***, **, * denote significant at the 1%, 5% and 10% levels respectively.

**Table 9:** Test results of lag order of panel VAR model.

Model	lag order	AIC	BIC	HQIC
(19)	1	0.503	2.716	1.384
	2	− 1.165**	2.067**	0.106**
	3	57.956	62.376	59.661
(20)	1	32.841	35.054	33.722
	2	30.472**	33.703**	31.743**
	3	69.465	73.885	71.169

Note: ***, **, * denote significant at the 1%, 5% and 10% levels respectively.

#### Analysis on the interactive effect of digitalization and traditional industrial upgrading in the Yellow River Basin

The in-group mean difference method and forward mean difference method were used successively to eliminate the time and individual effects respectively, and the numerical relationships among the study variables were estimated when the optimal lag period was 2 in Table 10, where the data in parentheses referred to the t statistic adjusted by white heteroscedasticity. According to the estimated results of model (19), at the significance level of 1%, the lag items of digital construction level and digital access level have a significant impact on the upgrading of 0.511 and 0.413 respectively on the traditional industrial upgrading, while the influence coefficient of digital circulation level and digital application level on the traditional industrial upgrading under the significance level of 1% is 0.003 and 0.001, respectively. Obviously, digital access level contributes the most to the traditional industrial upgrading, while digital circulation level and digital application level have little influence on the traditional industrial upgrading. Therefore, it is necessary to further strengthen the construction of digital infrastructure, increase the supply of network facilities and network services, and provide an important guarantee for the traditional industrial upgrading in the Yellow River Basin. In addition, the lag term of traditional industrial upgrading has the largest impact on the digital circulation level and the least impact on the digital access level. According to the estimated results of Model (20), at the significance level of 1%, the lag term of product upgrading and industrial ecological upgrading has a significant impact of − 0.098 and 0.085 on the digitalization level, respectively. Moreover, the lag term of process upgrading has a negative effect on the digitization level at the significance level of 5%, while the lag term of function upgrading has an insignificant negative effect on the digitization level. In addition, product upgrade, function upgrade and industrial ecological upgrade have significant effects on the digital level lag terms of − 0.410, 2.273 and − 0.119, respectively. In the above two models, digitalization level and traditional industrial upgrading are significantly affected by their own lag term at the significance level of 1%, which indicates that there is a certain inertia between digitalization level and traditional industrial upgrading^75^.

**Table 10:** System-GMM estimation results of panel VAR(2) model.

Model (19)	CSJ_t−2_	JS_t−2_	JR_t-−2_	LT_t−2_	YY_t−2_	Model (20)	SZH_t−2_	GY_t−2_	CP_t−2_	GN_t−2_	STH_t−2_
CSJ_t−2_	0.650*** (6.16)	13.070* (1.68)	0.562* (0.38)	18.817* (1.69)	13.577*** (3.65)	SZH_t−2_	1.066*** (6.53)	− 33.792* (− 1.17)	− 0.410*** (− 0.72)	2.273*** (1.22)	− 0.119*** (− 0.31)
JS_t−2_	0.511***(0.74)	0.310***(0.73)	0.175* (1.77)	1.672*** (3.07)	9.414** (2.01)	GY_t−2_	− 0.005** (− 1.96)	− 0.144*** (− 0.34)	− 0.040* (− 1.49)	0.093*** (2.85)	0.038*** (1.60)
JR_t−2_	0.413*** (4.34)	1.240 (1.53)	0.741*** (14.21)	1.410***(6.14)	2.214*** (17.55)	CP_t−2_	− 0.098*** (− 15.98)	1.157*** (0.59)	0.617*** (4.48)	0.392*** (7.58)	0.150*** (1.53)
LT_t−2_	0.003* (1.84)	0.174** (0.46)	− 0.007 (-0.27)	0.874***(7.12)	0.479* (1.65)	GN_t−2_	− 0.002 (− 0.16)	4.067*** (1.34)	0.566*** (5.41)	1.654*** (3.38)	− 0.099** (− 2.22)
YY_t−2_	0.001* (0.24)	0.155** (0.40)	0.171*** (3.10)	1.269** (2.49)	0.853*** (6.50)	STH_t−2_	0.085*** (1.51)	2.293** (2.42)	3.869* (1.70)	0.473** (2.30)	0.572*** (3.08)

Note: ***, **, * denote significant at the 1%, 5% and 10% levels respectively.

**Figure 6:**
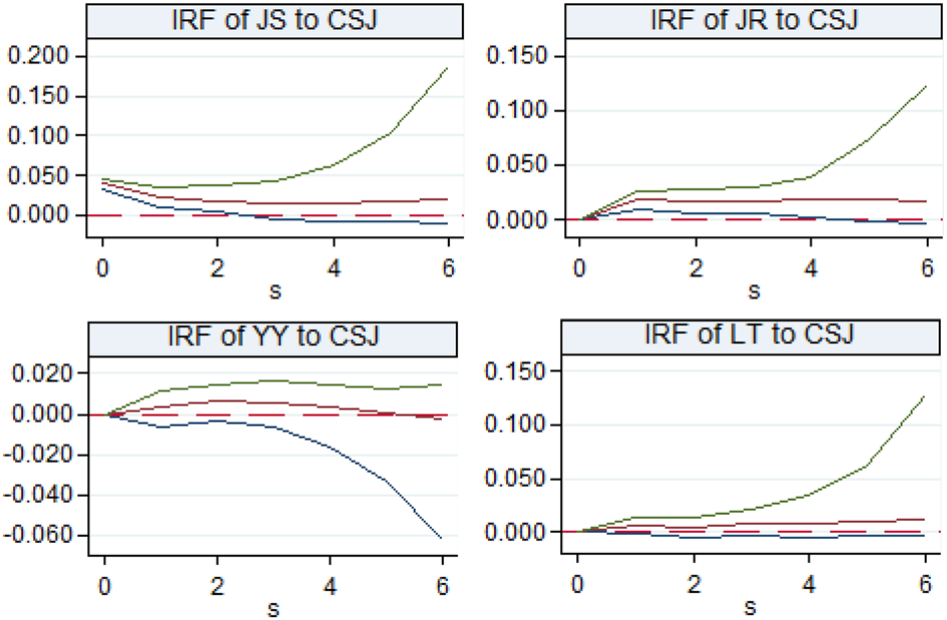
Impulse response to the impact of digitalization on traditional industrial upgrading in the Yellow River Basin.

**Figure 7:**
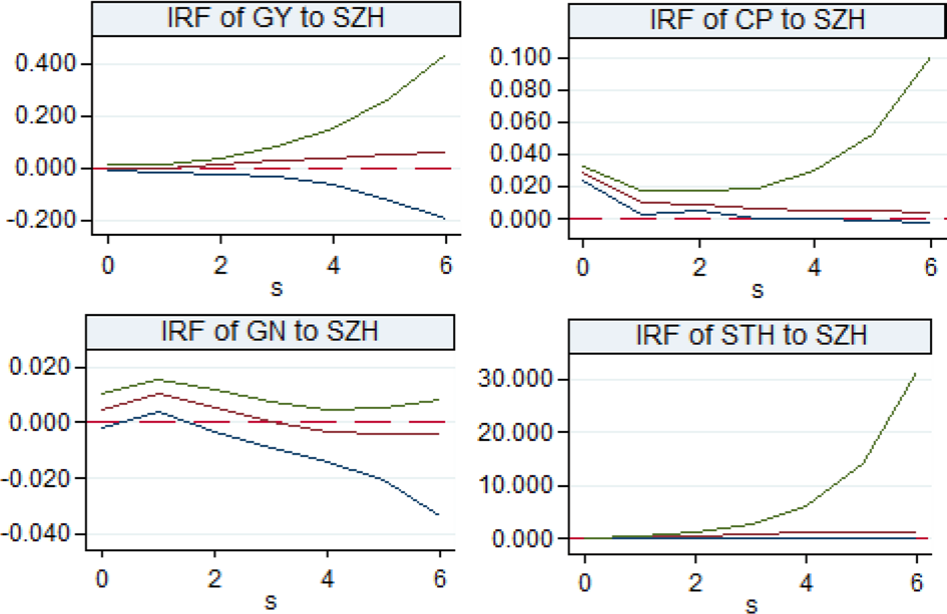
Impulse response of traditional industrial upgrading to digital impact in Yellow River Basin.

The impulse response function of panel VAR model can quantitatively describe the current and future impacts of an endogenous variable on other endogenous variables after applying an orthogonalization pulse of one standard deviation, and obtain the dynamic correlation features between the two endogenous variables while other endogenous variables are controlled. Furthermore, the dynamic interaction effect between digitalization level and traditional industrial upgrading can be more thoroughly observed by impulse response function graph based on the interaction characteristics of describing variables. The first step is to obtain the impulse response function by Cholesky decomposition. The second step is to run 300 simulations through Monte Carlo method to obtain a confidence interval of two standard deviations. The horizontal and vertical axes in Figs. 6 and 7 respectively represent the number of lag periods (s) and the impulse response value. The red and black solid lines represent the impulse response curve and the 95% confidence interval, respectively^76^.

1. Dynamic changes of traditional industrial upgrading in the Yellow River Basin under the impact of digitalization (Fig. 6). The impact of digital construction level on the traditional industrial upgrading has always been positive, and this positive influence shows a trend of convergence with the increase of the response period. It shows that the continuous improvement of digital construction level will be conducive to the upgrading of traditional industries in the Yellow River Basin. Traditionally, the level of digital construction refers to the degree of infrastructure perfection that can reflect the characteristics of the digital economy. These infrastructures are based on information networks and integrated with emerging digital technologies, which can provide a new zero-distance contact platform for technological progress and product innovation, accelerate the flow of resources between traditional industries, and enhance information transparency. In addition, digital infrastructure can collect, store and utilize data across multiple systems and equipment, provide necessary network resources for traditional industrial enterprises, strengthen the link between product supply and demand, provide information and technical support for enterprises to grasp user demand information, and develop products in a targeted manner, which will promote the upgrading of traditional industries.

The positive impact of digital access level and digital circulation level on the traditional industrial upgrading is 0 in the initial stage. The former immediately produces a positive response and tends to be stable after reaching a certain threshold, while the latter continues to increase with the continuation of time. It shows that the continuous improvement of digital access level and digital circulation level has a positive impact on the upgrading of traditional industries in the Yellow River Basin. On the one hand, the level of digital access refers to the level of network connectivity, which is usually manifested as the level of Internet broadband access or ICT access. Digital network access can help enterprises to manage production. Businesses with broadband Internet access can participate in a variety of online media activities, which can help foster more digital skills and enhance access to information resources. On the other hand, the level of digital circulation reflects the development degree of digital industrialization. Digital circulation is an important manifestation of consumption. The new digital circulation platform can change consumption patterns, promote consumption upgrading, assist enterprises to identify customer needs, improve resource integration and market circulation efficiency, and thus promote the upgrading of traditional industries.

The impact of digital application level on the traditional industrial upgrading is in a state of fluctuation. Specifically, the impact response was 0 in the initial stage, reached a peak in the second stage, turned negative in the fifth stage, and the negative impact increased steadily. It shows that the digital application level has both promoting effect and hindering effect on the upgrading of traditional industries. Among them, the level of digital application plays a dominant role in promoting the upgrading of traditional industries before the fifth phase. Digital application level refers to the degree of IT technology mastery and popularization. Compared with the extensive large-scale production mode of traditional industry, the cross-time, strong link and instantaneous characteristics of digital technology can resolve the conflict between production cost, product diversity, production cycle and other multi-objectives to a certain extent, so as to alleviate the problem of overcapacity in traditional industries with flexible production methods. Therefore, it will help promote the upgrading of traditional industries by accelerating the promotion of the digital application of traditional industries, promoting the digital transformation and intelligent upgrading of the production methods and organization methods of traditional industries. However, the negative effect of the digital application level on the upgrading of traditional industries has emerged after the fifth phase. It indicates that the problem of “Tool sprawl” of an enterprise becomes very serious when an enterprise’s application of digital technology is too advanced or employees must rely on more and more digital systems or tools to ensure the normal completion of their daily work. This phenomenon has a serious impact on data center operation, network security protection, system reliability and application performance. The hindrance to the upgrading of traditional industries will gradually exceed the promotion effect.

2. The dynamic change of digitization level in the Yellow River Basin under the impact of traditional industrial upgrading (Fig. 7). With the continuation of response time, the impact of process upgrading on digitalization level turns from 0 to positive, and the degree of positive influence is increasing. The impact from product upgrading always remains positive, but this positive influence gradually decreases to convergence with the continuation of time. The process upgrading of traditional industries refers to the improvement of production processes through the use of advanced production technology and production equipment. The most direct manifestation of product upgrading in traditional industries is that enterprises achieve promotion to both ends of the value chain by developing new products or transforming existing products. The process upgrading and product upgrading of traditional industries are conducive to promoting the spatial spillover of digital technology, promoting digital technology cooperation within and between industries, and effectively driving the digital development of other industries. At the same time, the optimization of traditional industrial structure can effectively promote the linkage development of upstream and downstream industries in the Yellow River Basin, and enhance the digitalization level of upstream industries.

The impact of industrial ecological upgrading on digitalization level turns from 0 to positive, and the degree of positive influence is increasing. Industrial ecological upgrading means that traditional industries choose production technologies with lower energy consumption and less pollution to optimize the allocation efficiency of energy in the process of production, so that traditional products continue to develop in the direction of low energy consumption and low pollution to achieve sustainable development. In the process of ecological upgrading of traditional industries in the Yellow River Basin, the production factors in the basin have shifted and the production efficiency has gradually increased, which will effectively improve the digital capability in the basin.

The impact from function upgrading starts from positive, and this positive impact decreases continuously until it turns negative and deepens in the third stage. After process upgrading and product upgrading, the industry has accumulated core technologies and the ability to develop new products. It will recombine and distribute the internal resources of traditional industries, gradually abandon low value-added links to produce high value-added links, in order to obtain the core competitiveness of traditional industrial enterprises and promote functional upgrading. Before the third phase, the technology spillover and promotion effect of the functional upgrading of traditional industries emerged, which could offset the cost effect of digitalization and help improve the digitalization level of enterprises. However, digital transformation is not a one-off project, but a continuous process of innovation. Many enterprises only carry out digital transformation in the early stage, lack of continuous investment and innovation, and eventually lead to the gradual weakening of the transformation effect in the later stage. The cost effect of digitization is higher than the compensation effect. Therefore, the overall effect was negative after the third stage.

Due to further analyze the structural impact of CSJ, JS, JR, LT and YY on CSJ in Model (19), and SZH, GY, CP, GN and STH on SZH in Model (20). The variance decomposition method is used to extract the more important information of variables affected by each random disturbance in this paper. According to the results of variance analysis in Table 11. CSJ, JS, JR, LT and YY all have a certain influence on CSJ in Model (19). On the whole, the contribution of CSJ shock to its own prediction variance is the largest and shows a decreasing trend. The peak contribution of JS, JR, LT and YY to CSJ reached 5.3%, 37.4%, 0.2% and 18.2%, respectively. In contrast, the contribution of JS to CSJ has a great impact in the short term, while the contribution of JR and YY to CSJ is stable in the long term, and the contribution of LT to CSJ has not been obvious. In Model (20), the contribution of SZH and GN shocks to the prediction variance of SZH firstly increased and then decreased, the contribution of GY shocks to the prediction variance of SZH maintained an increasing trend, while the contribution of CP and STH shocks to the prediction variance of SZH maintained a decreasing trend. The peak contribution of GY, CP, GN and STH to SZH reached 22.4%, 18.2%, 25.6% and 44.4%, respectively. Moreover, GY and GN make relatively large contribution to SZH. In addition, the interaction between digitalization and traditional industrial upgrading is asymmetrical, and the two contribute the most to their own impact value, which indicates that the “digitalization—traditional industrial upgrading” system presents significant positive feedback effect. Regions should reasonably allocate digitalized construction investment, digitalized access investment and digitalized application investment at different stages according to the actual situation, and avoid one-sided emphasis on digitalized construction investment.

**Table 11:** The results of variance decomposition.

Impacted variables		CSJ					SZH				
Shock variables		CSJ	JS	JR	LT	YY	SZH	GY	CP	GN	STH
Number of lag periods	1	0.923	0.053	0.003	0.000	0.021	0.356	0.022	0.135	0.042	0.444
	2	0.916	0.033	0.039	0.002	0.009	0.660	0.015	0.182	0.093	0.050
	3	0.894	0.016	0.062	0.002	0.026	0.609	0.023	0.115	0.227	0.026
	4	0.854	0.015	0.091	0.002	0.037	0.613	0.028	0.084	0.256	0.019
	5	0.830	0.014	0.108	0.002	0.046	0.656	0.038	0.059	0.234	0.014
	6	0.802	0.012	0.129	0.002	0.055	0.683	0.057	0.038	0.213	0.009
	7	0.771	0.009	0.152	0.001	0.066	0.680	0.086	0.023	0.205	0.006
	8	0.632	0.002	0.251	0.000	0.115	0.665	0.124	0.014	0.193	0.004
	9	0.632	0.002	0.251	0.000	0.115	0.642	0.171	0.008	0.175	0.003
	10	0.428	0.014	0.374	0.002	0.182	0.611	0.224	0.005	0.158	0.002

## Conclusions and Implications

### Conclusions

The research on the relationship between digitalization and traditional industrial upgrading has become a hot topic in academic circles. However, the research on the correlation between digitalization and traditional industrial upgrading in the Yellow River Basin is still at the theoretical level with different conclusions and relatively scattered. At present, China is in a critical period of industrial upgrading and the transformation of old and new driving forces in the economic structure. Moreover, digitalization has become an important starting point for promoting the high-quality development of China’s economy. In this context, clarifying the relationship between digitalization and the traditional industrial upgrading will help promote the upgrading of industrial structure in the Yellow River Basin and effectively achieve high-quality economic development in China.

In particular, in accordance with the provincial panel data of the Yellow River Basin from 2011 to 2020, this paper firstly constructed a coupling and coordination model of digitalization and traditional industrial upgrading. Secondly, the coupling coordination degree and relative development degree of interactive development are calculated, and the temporal and spatial evolution characteristics of the coupling coordination degree are analyzed. Thirdly, the panel VAR model of digitalization and traditional industrial upgrading in the Yellow River Basin is constructed. Fourthly, impulse response function and variance decomposition are utilized to further analyze the dynamic changes of traditional industrial upgrading under the impact of digital construction level, digital access level, digital circulation level and digital application level, and the dynamic changes of digitalization under the impact of process upgrading, product upgrading, function upgrading and industrial ecological upgrading.

The conclusions are drawn as follows:

1. The coupling coordination degree between digitalization and traditional industrial upgrading in the Yellow River Basin is continuously optimized. However, traditional industrial upgrading in the Yellow River Basin lags behind the digital level, and the two do not match. During the study period, the digitalization level, traditional industrial upgrading level and coupling coordination degree gradually increased, while the relative development degree gradually decreased. On the whole, the coupling coordination degree between digitalization and traditional industrial upgrading in the Yellow River Basin gradually experienced a transformation process of “disordered state → disordered state co-existing with low steady state → low steady state → low steady state co-existing with medium steady state”. At present, it is still in the stage of coexistence of low and medium steady state.
2. The coupling coordination degree of digitalization and traditional industrial upgrading is unbalanced and polarized among the nine provinces in the Yellow River Basin. Thereinto, Sichuan and Shandong have entered the running-in stage, while Qinghai, Gansu, Ningxia, Inner Mongolia, Shaanxi, Shanxi and Henan are still in the low-stable and antagonistic stage. In addition, there is a positive spatial correlation among the nine provinces. The provinces in the middle and lower reaches of the Yellow River showed a “high–high” concentration distribution such as Henan, Shanxi, Shandong and Shaanxi, while the provinces in the upper reaches of the Yellow River showed a “low–low” concentration distribution such as Ningxia, Gansu and Qinghai.
3. Both the digitalization and traditional industrial upgrading have self-reinforcing mechanisms, and there is a long-term dynamic correlation between them. In the long run, the improvement of the level of digital construction, digital access and digital circulation will improve the traditional industrial upgrading. The improvement of process upgrading, product upgrading and industrial ecological upgrading will increase the impact of digitalization level. However, in the short term, the improvement of the level of digital application will improve the traditional industrial upgrading, and the upgrading of functions will increase the impact of digital level.

### Implications

According to the conclusion 1, the transformation and traditional industrial upgrading in the Yellow River Basin is relatively backward, and the level of coupling and coordination between digitalization and traditional industrial upgrading is not high. On the basis of clarifying the development pain points of traditional industries in different regions of the Yellow River Basin, the government should improve laws, regulations and institutional systems as soon as possible to reduce the cost of digital transformation of traditional industrial enterprises. First, the government should provide financial subsidies or tax incentives for traditional industrial enterprises' investment in digital transformation to encourage enterprises to accelerate the pace and intensity of digital transformation. Second, the government should guide government funds and other types of social capital to actively support the development of the digital economy, grant project funding to enterprises that independently research and develop digital software, and promote the key research and pilot application of industrial software technology. Third, the government should speed up the construction of digital integrated application platforms. With the driving and supporting role of digital high-tech such as artificial intelligence, blockchain, cloud computing, big data, the Internet of Things, and 5G, high-quality development of traditional advantageous industries such as transportation and manufacturing in the Yellow River Basin will be promoted. For traditional industrial enterprises, on the one hand, they should focus on promoting the application of digital technology, and practice the concept and application of digital management in the whole process of product design, production planning, manufacturing process, organization, service and so on. In addition to themselves, they should also pay attention to promoting the transformation and upgrading of upstream and downstream enterprises, and ultimately achieve the double upgrading of the industrial chain and value chain. On the other hand, it is necessary to strengthen digital vocational training for talents needed in various industries, standardize the evaluation criteria for digital talents, accelerate the formation of a talent team with digital knowledge, diversified structure and reasonable level to adapt to the development of the digital economy, and constantly improve the data utilization efficiency and talent training efficiency of traditional industries for digital transformation.

According to the conclusion 2 and conclusion 3, there are significant differences in the coupling coordination degree between digitalization and traditional industrial upgrading among provinces in the Yellow River Basin. On the one hand, the developed provinces and regions should play a leading role in improving the coupling coordination level of digitalization and traditional industrial upgrading in the Yellow River Basin. At present, Sichuan and Shandong are the two major provinces in the Yellow River Basin, and their driving role is not obvious, which seriously affects the collaborative upgrading mechanism of the coupling level of digitalization and traditional industrial upgrading between provinces in the Yellow River Basin. The Yellow River basin should gradually strengthen the advantages of large cities. The first is to promote the development of three state-level urban clusters which are namely the Shandong Peninsula, the Central Plains and the Guanzhong. The second is to give full play to the guiding role of leading cities such as Zhengzhou, Qingdao, Jinan and Xi 'an. On the other hand, there is a positive spatial correlation between the coupling coordination levels in different regions. Therefore, the government should focus on improving the reality that the spatial spillover effect of digital and traditional industrial upgrading is weak between provinces, and strengthen the demonstration effect of digital development and the spillover effect of industrial chain in the provinces within the basin. Specifically, the government can combine the resource endowment conditions, social market needs and technological conditions of different regions. First, the government should accelerate the effective flow and efficient agglomeration of regional production factors, increase regional interactive digital sharing centers, and build a mutually beneficial digital ecosystem. Second, the government can improve the digital economy industrial chain and promote digital integration in the Yellow River basin. Third, governments can jointly improve the efficiency of regional resource allocation, enhance the value of traditional industrial chains, and guide the digital development of traditional industries. For example, due to help traditional industries expand functions, reduce costs, improve efficiency, and increase power, it is necessary to build a digital service platform of industrial interconnection and global awareness in combination with the regional industrial layout and provide a supporting platform for the high-quality collaborative development of traditional industries in the provinces of the Yellow River Basin.

Although the relevant viewpoints proposed in this paper are supported by theoretical and practical data, there are still some areas to be improved due to the time and level of research. For example, the research dimension of this paper is limited to the Yellow River basin, and it is necessary to carry out comparative studies on other important economic zones in China, such as the Beijing-Tianjin-Hebei region, the Yangtze River Economic Belt, the Guangdong-Hong Kong-Macao Greater Bay Area, and the Yangtze River Delta. In addition, the factors affecting the relationship between digitalization and traditional industrial upgrading are not fully considered. For example, the coupling and coordinated development of the two is also affected by many other factors such as government regulations. Future research should focus on more influencing factors.

## Data Availability

The datasets used and/or analysed during the current study available from the corresponding author on reasonable request.
